# Editorial: Beyond psilocybin: exploring the clinical potential of alternative and novel psychedelics

**DOI:** 10.3389/fpsyt.2025.1600812

**Published:** 2025-04-23

**Authors:** Martin L. Williams, Deborah Rudin, Yasmin Schmid, Jacqueline von Salm

**Affiliations:** ^1^ Centre for Mental Health & Brain Sciences, Department of Psychology, Swinburne University of Technology, Hawthorn, VIC, Australia; ^2^ Psychedelic Research in Science & Medicine (PRISM), Melbourne, VIC, Australia; ^3^ Clinical Pharmacology and Toxicology, Department of Biomedicine and Department of Clinical Research, University Hospital Basel, Basel, Switzerland; ^4^ Department of Pharmaceutical Sciences, University of Basel, Basel, Switzerland; ^5^ Psilera Inc., Tampa, FL, United States

**Keywords:** psychedelic medicine, psilocybin, 5-MeO-DMT, DMT, ayahuasca, psilacetin, psychedelic-assisted therapy, LSD

Psychedelic research has flourished over the past fifteen years (1, 2), following several decades in the political – and medical - wilderness. Psilocin, generally administered as its prodrug, psilocybin, is seen as a prototype of the so-called “classic psychedelics”, playing a leading role in recent clinical trials investigating psychedelic-assisted psychotherapy for the treatment of a range of mental health conditions (3, 4), as well as fundamental research into brain function and consciousness (5).

Strategically, the current wave of psychedelic research commenced with psilocybin primarily because it was relatively unfamiliar both to regulators and to the public and hence lacked the sociopolitical baggage of the potent and widely known trailblazer, LSD (6). Psilocybin also offered the perceived advantages over LSD of being a natural product with a relatively short duration of action and a lower potential for adverse psychological effects in therapeutic contexts (7).

However, psilocybin is but one of scores of compounds belonging to two main chemical classes – phenethylamines and tryptamines - along with ergolines and an expanding array of other (mostly heterocyclic) alkaloids - that act as agonists of the serotonin 5-HT_2A_, 5-HT_2C_ and 5-HT_1A_ receptors responsible for the various phenomenological and putative therapeutic effects of classic psychedelics (8). In addition, attention is turning to other compounds within the broader class now known as “psychoplastogens”, that do not elicit the characteristic phenomenological effects associated with psychedelics but nonetheless show promise as pharmacotherapeutic agents through promotion of neuroplasticity (9).

While psilocybin has proved to be an excellent “gateway psychedelic” for clinical research, showing efficacy in the treatment of a range of mental health conditions, a range of properties – physicochemical, pharmacological, and phenomenological - could be varied and optimized to extend the application of psychedelic therapies to broader patient populations and conditions.

Thus, there is clear potential to be explored among the many alternatives to psilocybin. These include legacy psychedelics such as mescaline, *N*,*N*-dimethyltryptamine (*N*,*N*-DMT), and 5-methoxy-*N*,*N*-dimethyltryptamine (5-MeO-DMT), plus a range of analogs created in the latter half of the 20^th^ century and a suite of entirely novel compounds emerging more recently through the efforts of a new generation of researchers working in academia and within a burgeoning community of small biotech enterprises.

In launching this Research Topic, we believed the time to be ripe for a focused discussion of the intrinsic interest and therapeutic potential of psychedelics that exist “beyond psilocybin”. Consequently, seven articles were published in this Research Topic, covering perspectives, preclinical research, and human studies of established and emerging psychedelics for the treatment of mental health conditions ranging from depression and post-traumatic stress disorder (PTSD) to anxiety in the context of palliative care.

Two case reports kick off the Research Topic. Reissmann et al. present a single-case study of a personalized dose regimen of repeated ketamine infusion for the treatment of major depressive disorder, discussing some of the insights gained in the context of maintaining altered states of consciousness (ASCs). Noting the lack of published research investigating possible associations between ASCs and the antidepressant properties of ketamine, the authors query whether the persistence of recurring ASCs in the course of repeated ketamine infusions is necessary for the maintenance of its demonstrated antidepressant effects.

Ragnhildstveit et al. provide a report of their longitudinal study of the treatment of PTSD in one patient using vaporized 5-MeO-DMT. This compound is emerging as a promising classic psychedelic that offers the potential benefits of short duration of acute effects and a somewhat distinct pharmacological profile that features 5-HT_1A_ receptor agonism over the 5-HT_2A/2C_ activity predominantly exerted by psilocybin and other classic psychedelics. While much remains to be elucidated through clinical studies currently underway, 5-MeO-DMT may hold a key to the exploration of subtle differences among classic psychedelics for the treatment of mental health conditions.

A comprehensive article by Tasker et al. reviews the subclass of clavine alkaloids in the context of the complex pharmacology of related ergolines, including their most historically noteworthy exemplar, LSD. While lacking the carboxyl group of the lysergic acid amides, the closely related clavines nonetheless display significant activity at the 5-HT_2_ receptors of interest to psychedelic research, alongside affinities to a broad range of monoamine and other receptors that contribute to the fascinating pharmacology of ergot alkaloids and their derivatives.

Moving to clinical trials, the report by Aicher et al. of their Phase 1 RCT of an ayahuasca analog in healthy subjects details acute and persisting effects following the administration of harmine (a reversible inhibitor of monoamine oxidase A, MAO-I) as orodispersible tablets, followed by intranasally administered DMT at incremental dosages. While administration of an MAO-I facilitates the oral activity of DMT, it also extends the duration of the effects of DMT when administered by parenteral routes. This early-phase trial in healthy humans leads the way to future trials of this and similar approaches to administering DMT to clinical populations.

Meanwhile, James et al. report on an industry-led study of a novel DMT fumarate formulation in healthy participants, likewise leading the way to future trials in clinical populations. Without the inhibitory effects of an MAO inhibitor such as harmine, DMT must be administered parenterally in order to elicit psychedelic effects, so this study explored the effects of intravenous administration of a novel DMT formulation in terms of safety, tolerability, impacts on mood, and pharmacodynamics to inform key elements of a subsequent Phase 2a study of this formulation in the treatment of major depressive disorder.

Staying true to our theme, “Beyond Psilocybin”, Jones et al. present results of their preclinical PK study of psilacetin, a currently unscheduled prodrug of psilocin that, when administered in the fumarate form to mice, appears to yield a lower peripheral psilocin exposure *in vivo* than that following psilocybin administration, and thus may provide some advantages in terms of dose titration in further animal studies and, potentially, in future human trials. In the meantime, psilacetin is proposed by the authors to be a useful research compound in preclinical psychedelic studies, thanks to its (currently) unscheduled status.

Finally, we present a manuscript by Wang et al. that addresses the very promising application of classic psychedelics to the treatment of end-of-life distress in the context of palliative care and palliative care patients’ attitudes towards this treatment option. This article discusses a range of themes that we anticipate will apply much more broadly as research and clinical communities continue to explore applications of psychedelics beyond psilocybin to the treatment of mental health conditions.

The manuscripts included in this Research Topic reflect the renewed global interest and ongoing progress in psychedelic clinical research, a fascinating area that, with some justification, is being described as a new paradigm in mental health treatment. While psilocybin has served as an excellent starting point for psychedelic research, we are encouraged to see the broadening of scope to explore a much wider range of psychedelic compounds, both already existing and yet to be created.

It is hoped that this Research Topic will spark interest and ultimately add further richness to the dynamic field of psychedelic research.
